# Efficient high-volume cataract services: the Aravind model

**Published:** 2014

**Authors:** Thulasiraj Ravilla, Dhivya Ramasamy

**Affiliations:** Executive Director: Lions Aravind Institute of Community Ophthalmology, Aravind Eye Care System, Madurai, India. thulsi@aravind.org; Faculty Associate: Lions Aravind Institute of Community Ophthalmology, Aravind Eye Care System, Madurai, India. dhivya@aravind.org

**Figure:**
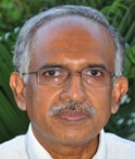
Thulasiraj Ravilla

**Figure:**
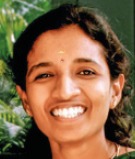
Dhivya Ramasamy

Aravind Eye Care System began as an 11-bed eye clinic in 1976. Over the last 36 years, over 40 million outpatient examinations have been performed and over 5 million patients have undergone eye surgery or laser procedures.

Aravind, with its mission to ‘eliminate needless blindness’, has been able to achieve this by adhering to the principle of providing large volume, high quality and affordable services in a financially sustainable manner both for the patients and for Aravind. Much importance is given to equity – ensuring that all patients are accorded the same high quality care and service, regardless of their economic status.

A critical component of Aravind's model is the high patient volume, which brings with it the benefits of economies of scale. This article talks about how Aravind built this high patient volume and kept the costs low to provide efficient, good quality and affordable eye care.

## Building patient volumes by reaching out into the community

Most hospitals tend to focus only on patients who seek care from them or have systems to attract those who would otherwise go to other hospitals. In a situation where less than 15% of the people who needed eye care were seeking it, Aravind chose to focus on the other 85% – the ‘non-customers’ – and to build systems and processes to reach them.

Aravind does this by reaching out into the community through active partnerships with social organisations, local philanthropists, volunteers, the school system and industries in the local community.

Outreach screening camps are organised to reach out to the general population, school children and industry workers. These camps are visited by over half a million people each year, of whom over a third receive some significant intervention. Overtime, the camps enhance the public's awareness of eye care and improves the health-seeking behaviour in the community, thereby growing the customer base for the hospital.

**Figure:**
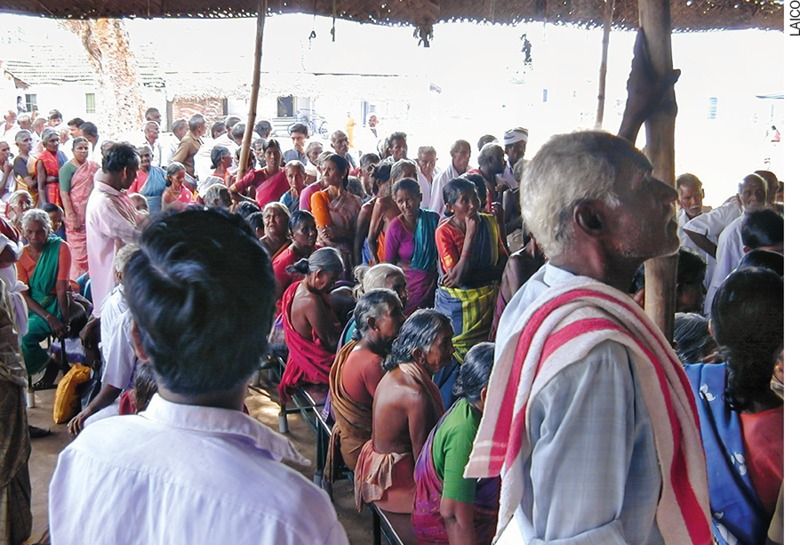
Registration in process at an Aravind eye camp

About 2,500 outreach screening camps are conducted each year and these are highly standardised, resulting in increased efficiency and lower case finding costs. The involvement of local community groups helps to build patient confidence and makes use of local resources, thereby reducing the cost even further.

More recently, a network of 45 vision centres were established, with each centre covering a population of 60,000–70,000. Patients ‘walk in’ of their own accord and are referred to the ‘base hospitals’ if they need cataract surgery. Through this combination of community partnered outreach and vision centres, Aravind performs over 100,000 cataract operations each year. As a result of the awareness created through outreach and other means, another 150,000 cataract operations are done on patients who come to the base hospitals of their own accord.

## Reducing the cost to the patient

A key factor influencing uptake of services is affordability. This is best addressed by a holistic perspective which takes into account the total costs incurred by the patient, of which the hospital charges are a part. Besides ensuring that the hospital charges are affordable to the patient (which is a significant influence on demand), Aravind also addresses the other costs incurred by the patient in the way the services are designed and provided.

The following strategies to reduce travel and the associated costs and effort (what we call ‘patient costs’) have been employed:

- Eye care is made locally available through outreach and vision centres, which greatly reduces travel and associated costs.
- All investigations are made during a single visit, eliminating the need for the patient to make multiple visits and thus reducing travel and associated costs.
- Patients are offered a surgery slot immediately if surgery is indicated. There is no waiting list. This enables patients to complete the entire care cycle in a single visit.
- Prescribed medicines or spectacles are made available locally and at a fair price.
- Free transportation is offered to all patients identified during outreach as needing surgery. The patients are accompanied to the hospital and back by Aravind staff or a community volunteer. This costs less than a third of what it would cost if the patients were to come on their own, because they would otherwise have to pay their own transport and that of their accom-panying person. Also, because Aravind transports several patients at a time, they are able to charter a bus for the trip and the cost per person is less than if people travelled as individuals.

## Reducing provider costs

The costs associated with providing patient care (the ‘provider costs’) can be reduced by organising the system more efficiently, eliminating errors, and ensuring quality in all that is done.

First, Aravind recognised that ophthalmologists are scarce and expensive. Over 80% of the tasks that they performed were routine and repetitive (e.g. measuring intraocular pressure and assessing refractive error). Some of them, like prepping the patient for surgery, were time consuming. Aravind shifted these tasks to a new cadre of personnel broadly known as mid-level ophthalmic personnel. By deploying these personnel in the right proportions, it was possible to more than quadruple the productivity of a single ophthalmologist.

Developing standardised protocols, checklists and a quality assurance process also ensured that all required tasks – including patient communication – were performed at the right time and in the correct manner every time. Doing so systematically minimised errors, reduced costs and built patient confidence.

## Empowering patients for better compliance

Aravind chose to address patient compliance by developing a cadre of staff called patient counsellors whose main task was to make sure that the patient fully understood the importance of whatever was prescribed – surgery, medication, spectacles or follow-up. Where appropriate, the patients are reminded by phone or SMS. Aravind has over 200 such patient counsellors at a ratio of one counsellor for every two ophthalmologists.

The eye care delivery systems are designed to make compliance easy. For instance, the six-week surgical follow-up examinations for outreach patients are done at the camp site itself, making it very easy for the patient to comply.

Compliance is closely monitored and improvements are made to the counselling process or other systems, as required, on an ongoing basis.

## Replicating the model

The Lions Aravind Institute for Community Ophthalmology has worked with over three hundred hospitals across India and with thirty other countries – spanning Asia, Africa and Latin America – to help them replicate the principles behind the Aravind model. In brief, for an eye hospital to be effective under the Aravind principles, it should:

- reach out and serve all those in need
- use scarce resources optimally
- ensure that service outcomes are good
- develop financial viability for the hospital through patient revenues.

**Figure:**
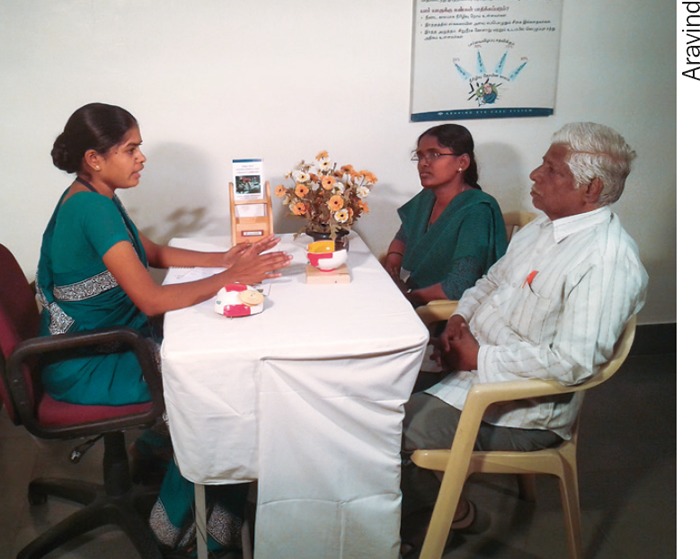
A counsellor speaks with a patient and his accompanying person

These principles make for a true win-win situation – better value for the patients while strengthening the hospital. For this to work, the principles have to be carefully balanced. For example, financial viability can't be achieved by charging unaffordable prices since that will negate the principle of reaching everyone in need. Also, good outcomes, and hence patient satisfaction, cannot be achieved unless the scarce resources are set up to serve the needs of the patients in a patient-centric system. Our understanding of productivity – and success – has to take into account all of the above.

Aravind, which is located in south India, had to consider the area's dense population, reasonable roads, good public transport and large middle class when it applied the principles behind the Aravind model to the design of its pricing structure and specific processes for generating demand. Similarly, in other countries and settings, the systems, processes and pricing structures must be relevant to the local demographic, social-economic and cultural realities. As these realities vary very widely, this is a challenge that may require some innovative thinking. Aravind therefore offers a systematic mentoring process to those hospitals who want to replicate the Aravind model.

The mentoring process starts with a detailed site visit to help the Aravind team understand the local realities such as population density, service area characteristics, local economy, hospital resources (including human resources), current performance levels, governance structure and management systems. This forms the basis for guiding the hospitals to develop the systems needed to generate demand (with a focus on people who wouldn't normally use the services – the ‘non-customer') and to support efficient workflow, quality assurance and a transparent fee structure that includes fee waivers for those who cannot pay. Specific organisational capabilities and individual competencies have to be in place to make these systems work well. The hospital's leadership team is offered opportunities to build their organisation's capabilities through training programmes, workshops and problem solving, as well as access to online resources.

The impact and result of this process, as can be expected, is quite varied. Overall, the hospitals mentored by Aravind have shown a 40–50% increase in their output (number of operations) in the year immediately following the replication process; in many instances the output doubled (a 100% increase) in the year thereafter. The cost recovery (the extent to which the recurring costs, not the capital expenses, were met through service fees) went up from 60% to 90%. Such overall figures can, however, be misleading, as they also include data from hospitals in which the process did not work. The real lessons in understanding the challenges come from these.

In hospitals where the leadership team treated the change process as a time-limited project, and was also deeply engaged, the improvements were quick, significant and sustained. In instances where the leadership team was distanced from the hospital and thus not engaged, the impact or improvements were marginal. Because achieving financial viability also has policy implications, this was not possible in some government-run facilities and some charitable hospitals, whether because of their policies or because of their charter.

The overall lesson from carrying out this replication process in over three hundred eye hospitals in varied settings is that the broad principles are universally applicable. However, the way these are translated into systems and processes must take into account local realities. Even with well-designed, locally relevant systems, the desired impact is achieved only when the systems are implemented well. This is entirely dependent on the quality of leadership and the leadership team's ability to provide the resources needed to support the change process.
